# Literary Notes

**Published:** 1895-12

**Authors:** 


### Literary Notes.

The Complete Farrier and British Sportsman is the title of a curious old quarto volume owned by Mr. George Urban, Jr., of Buffalo, and which we have had the privilege of examining. The author is Richard Lawrence, veterinary surgeon, and it is published by Thomas Kelly, 17 Paternoster Row, London, 1833. It announces on its title page that it is a systematic enquiry into the structure and animal economy of the horse, the causes, symptoms and most approved methods of prevention and cure of all the diseases to which he is liable. It includes a faithful delineation of the various dogs used in the sports of the field, with canine pathology. It is interspersed with sporting anecdotes and an account of the most celebrated horses and dogs, and is embellished with a series of engravings executed by eminent artists from original drawings in the possession of noblemen and gentlemen of the turf.

The volume is full of interesting reading and shows the status of veterinary science sixty years ago.

---

The *New Orleans Medical and Surgical Journal* for November published our obituary notice of Louis Pasteur in full, courteously giving us due credit.

In marked contrast, the *American Journal of Ophthalmology* published our obituary notice of Dr. Henry W. Williams in full in its October number, for which it did not give us credit. Lest the vision of our ophthalmic contemporary may be defective we respectfully invite its attention to the fact that this journal is copyrighted.

---

The Hodgkin Prize Paid.—J. R. Roosevelt, secretary to the United States embassy, London, has presented to Lord Rayleigh and Professor Ramsay the check of the embassy for $10,000, being the Hodgkin prize awarded by the Smithsonian Institution, of Washington, for their discovery of new properties in the atmosphere. The recipients of the prize have written a letter of thanks to the Smithsonian Institution.

---

The *Ladies' Home Journal* has set an example which our so-called religious papers should, but will not, follow. This magazine, one of the most widely circulated of all American periodicals, declines all advertisements of a "medical, remedial, or curative nature,"

from an unwillingness to assume any degree of sponsorship for anything that might be improperly suggestive or that, used by the ignorant, might do harm. It would be a relief, indeed, to pick up a church paper in which one could read without encountering the advertisements of all kinds of charlatans with their pills "for the regulation of" this or that and the suggestive "removal of obstructions," and others containing advertising lies which the editors of the papers cannot fail to recognise as lies. Did you ever read the denunciation of a medical fraud in a religious newspaper? —*Ohio Medical Journal.*

---

THE Alvarenga prize for 1895 has been awarded to Dr. Guy Hinsdale, of Philadelphia, for his essay entitled Syringomyelia. The next award of the Alvarenga prize, amounting to about $180, will be made on July 14, 1896, provided that an essay deemed by the committee of award to be worthy of the prize shall have been offered.

Essays intended for competition may be upon any subject in medicine, but cannot have been published, and must be received by Dr. Charles W. Dulles, the secretary of the college of physicians, on or before May 1, 1896.

---

THE College of Physicians and Surgeons, of Chicago, through its secretary, Dr. Bayard Holmes, has made an offer to the Women's Christian Temperance Union, which, in substance, is to erect a $150,000 building under such conditions that payment will be easy. The invitation to accept the offer came at a reception tendered Miss Frances Willard by the board of managers of the National Temperance Hospital. The proposition from the college is to furnish the W. C. T. U. with a hospital, which shall be conducted under its auspices and on the same lines as is the hospital now under its management. A committee has been appointed to consider the proposition and report at an early date on the feasibility of its acceptance.

---

THE state lunacy commission has made provision for the publication of a quarterly medical journal, to be issued from the Utica State Hospital, beginning in January, 1896. The contributors are to include the superintendents and assistant physicians in the state hospitals for the insane.

---

MESSRS. LEHN & FINK have issued a well-printed brochure of twenty-eight pages, giving interesting and valuable information concerning antitoxins, vaccine virus and other biological products of the biological and vaccinal department of the New York Pasteur Institute. It contains clinical reports on results obtained from these products as well as detailed directions as to dosage and treatment, besides much other information of value relating to these products. Physicians addressing the above firm, P. O. Box 3083, New York City, may obtain the pamphlet free.

---

THE *Medical and Surgical Register* of the United States, published by R. L. Polk & Co., Detroit, Mich., is about to make its fourth appearance on the same general lines of construction as heretofore. It will, however, be considerably enlarged and brought forward to cover the necessities of the present. It is the most useful medical register extant.

---

PEDIATRICS, a new journal owned by Dr. Dillon Brown, of New York, and edited by Dr. George A. Carpenter, of London, has made its first appearance. It is published twice a month by the Van Publishing Company, of New York.

---

THE *Cleveland Medical Gazette* begins a new volume with its November issue, 1895, in a new dress. It is enlarged in size and every way improved in appearance with a view to keep pace with the progress of the age.

---

THE *Canada Medical Record* has put on a new dress and reduced the size of its page to standard octavo. The new editor-in-chief, Dr. J. Bradford McConnell, seems to be infusing the *Record* with new energy.

